# Small-Scale School-Based Cancer Education to Improve Awareness and Risk Reduction Knowledge Among Adolescents: A Pilot Study

**DOI:** 10.3390/ijerph23070823

**Published:** 2026-06-23

**Authors:** Nia Imani Bailey, Jenna Bucolo, Katelyn Bucolo, Brittnee Cannon, Samuel Elenwo, Monique Gary, Trudean Haye, Rebecca Kusters

**Affiliations:** 1 Byrd Cancer Education and Advocacy Foundation, Drexel Hill, PA 19026, USA; jenna.canale@pennmedicine.upenn.edu (J.B.); katelyn.e.bucolo@christianacare.org (K.B.); selenwo@pa.gov (S.E.); 2 Your Plant Based Baby, Philadelphia, PA 19406, USA; brittnee@yourplantbasedbaby.com; 3 Still Rise Farms, Perkasie, PA 18944, USA; info@stillrisefarms.com; 4 ITAVFoundation, Philadelphia, PA 19138, USA; info@itavf.org; 5 Live Like Lukas Inc., Newark, DE 19808, USA; livelikelukasinc@gmail.com

**Keywords:** small-scale cancer education, pilot study, school health, adolescents, health literacy, prevention

## Abstract

**Highlights:**

**Public health relevance—How does this work relate to a public health issue?**

- This pilot study addresses an important public health concern within the United States: rising cancer incidence among adolescents and young adults, combined with low awareness of modifiable risk factors.
- It identifies substantial gaps in students’ baseline cancer-risk reduction knowledge, including the importance of vaccination (21%), risk from smoking and alcohol (45%), and symptom recognition (51%).

**Public health significance—Why is this work of significance to public health?**

- Using a pre–post design, our study demonstrates that two separate, structured educational sessions can produce meaningful improvements in cancer knowledge, awareness of modifiable risk factors, genetic literacy, and understanding of risk reduction strategies, including HPV vaccination and sun safety.
- These findings suggest that even small-scale early education targeting cognitive precursors to behavior change may be an effective strategy for reducing long-term cancer risk and addressing health literacy disparities.

**Public health implications—What are the key implications or messages for practitioners, policy makers and/or researchers in public health?**

- Integrating cancer education on a small scale into school health curricula represents a feasible, scalable strategy to promote risk reduction, early detection, and health equity across populations.
- Policymakers and practitioners should prioritize scalable investment in school-based, risk reduction-focused education and caregiver engagement to reinforce health knowledge and support long-term behavior change.

**Abstract:**

Cancer incidence among adolescents is increasing, yet cancer risk reduction education remains largely absent from school-based curricula. This pilot study assessed whether a small-scale early, developmentally appropriate intervention could improve cancer literacy to support long-term risk reduction. This pilot study used a convergent parallel mixed-methods pre–post design to evaluate two separate, 45 min, school-based cancer education interventions delivered to 24 middle-school students in Pennsylvania. The intervention delivered developmentally appropriate content on cancer biology, modifiable risk factors, genetics, HPV vaccination, and self-advocacy using a low-resource, low-investment model easy for schools to implement. Pre- and post-intervention surveys assessed student knowledge, awareness, and health-related perceptions. Survey data were analyzed both descriptively using frequencies and percentages and thematically. Post-intervention results demonstrated substantial improvements across all domains. Correct definition of cancer increased from 16% to 100%. Awareness of modifiable risk factors increased to 96%, sunscreen knowledge to 90%, genetic testing awareness to 83%, and HPV vaccine understanding from 21% to 57%. Students also reported increased confidence in recognizing symptoms and engaging in health-seeking behaviors. Findings suggest that small-scale, school-based cancer education interventions are feasible and effective in improving adolescent cancer literacy. These results support the need for larger, controlled studies to evaluate long-term knowledge retention and behavioral outcomes.

## 1. Introduction

Cancer remains a leading cause of morbidity and mortality worldwide, and its incidence is increasing among adolescents and young adults [1]. Although cancer is often perceived as primarily genetically driven, fewer than 10% of cases are attributable to hereditary factors, while approximately 40% are considered preventable through modifiable lifestyle and environmental behaviors [1,2,3,4]. The American Institute for Cancer Research Cancer Risk Awareness Survey, for example, found that only about half of U.S. adults recognize established links between cancer risk and alcohol consumption, poor dietary patterns, insufficient physical activity, and excess body weight [3]. Epidemiological evidence demonstrates that dietary patterns, physical activity, and weight status play significant roles in the development of several common cancers, including colorectal, breast, and prostate cancer [2,4,5]. Similarly, the rapid increase in early-onset colorectal cancer has occurred too quickly to be attributable solely to inherited risk, underscoring the influence of modifiable factors [2,4].

The rising incidence of cancer among younger populations especially represents a growing public health concern that cannot be explained by genetic predisposition alone. Population-level surveillance data demonstrate increasing rates of several cancers among adolescents and young adults, including breast and colorectal cancers—trends that have occurred too rapidly to be attributed to inherited genetic risk alone [2,4]. While familial cancer syndromes play an important role for a small subset of individuals, the majority of cancers arise from modifiable environmental, behavioral, and social determinants of health, underscoring the limitations of genetic explanations in accounting for observed incidence patterns [6].

Persistent misconceptions regarding cancer etiology, combined with limited exposure to risk reduction-focused education, contribute to gaps in health literacy that may delay risk-reducing behaviors and early detection efforts across the life course. Research consistently demonstrates low awareness of cancer risk factors, warning signs, and risk reduction strategies among adolescents, as well as barriers to timely help-seeking [1,4]. These knowledge gaps have been associated with diagnostic delays and poorer outcomes, even for cancers where early detection is possible [5,7].

Educational interventions targeting school-aged children offer a critical opportunity to address these gaps [1,4]. Adolescence is a formative developmental period during which health beliefs, behavioral patterns, and self-advocacy skills are established and often persist into adulthood [7]. Introducing cancer education during this stage may normalize risk reduction, improve health literacy, and support sustained engagement in risk-reducing behaviors, including symptom recognition and timely care-seeking [3,8].

Risk reduction strategies—particularly those related to nutrition, physical activity, and healthy environments—are central components of comprehensive cancer control [2,4,5]. For example, excess body weight during childhood is associated with chronic inflammation, insulin resistance, and altered hormonal regulation, all of which are linked to increased cancer risk later in life [2,4]. Conversely, plant-forward dietary patterns are consistently associated with lower overall cancer risk [5]. Establishing healthy behaviors such as healthy eating and exercise early may meaningfully reduce cumulative lifetime exposure to modifiable cancer risk factors [2,4].

In addition to risk reduction, education about cancer signs and symptoms is essential. Early recognition of bodily changes and timely health-seeking behaviors may contribute to earlier diagnosis and improved outcomes. Lived experience from pediatric cancer advocacy underscores the importance of early education. Pediatric cancers frequently present with nonspecific symptoms such as pain, fatigue, or swelling, which can delay diagnosis and worsen outcomes when early signs are not recognized [6,9,10]. One good example of this can be seen in the primary author’s pediatric work with Rebecca Kusters, founder of Live Like Lukas, Inc., in producing pediatric cancer education. This community-centered work draws on the Kuster and Bailey’s first-hand lived experiences with pediatric cancer patients, emphasizing that developmentally appropriate education focused on body awareness and communication may empower children, caregivers, and educators to recognize when medical evaluation is warranted [10]. Schools therefore represent a uniquely positioned and equitable setting for risk reduction-focused cancer education.

From an equity lens, early access to cancer education may address disparities in health literacy that contribute to inequitable cancer outcomes across the life course [1,3,4]. Students in under-resourced schools, who often face limited access to risk reduction health information and healthcare services, may experience even greater deficits. In such contexts, students’ understanding of cancer is often shaped by personal or familial experiences, which can contribute to misinformation, fear, and missed opportunities for risk reduction and early detection [1,3,4,11]. Embedding cancer education into curricula may serve as an equalizing strategy, ensuring all students have access to foundational risk reduction health knowledge regardless of their resources outside of school [4,7].

Despite this evidence, much of cancer-related funding remains focused on treatment rather than risk reduction. National Cancer Institute data indicate comparatively limited investment in scalable, school-based prevention initiatives [8,12,13]. Qualitative research indicates that adolescents value cancer risk reduction and recognize that preventive behaviors can reduce risk; however, formal cancer education remains limited in many school curricula [2,4,13]. One reason for this might be the associated costs in resources, time, and financial implications of designing and implementing large-scale curricular interventions. Research indicates that logistical and system-level constraints significantly hinder the implementation and scalability of cancer and health education initiatives in school settings. Schools often face limited institutional capacity, insufficient coordination across stakeholders, and competing academic priorities that reduce the time and emphasis placed on health-related programming. In addition, practical barriers such as inadequate funding, staffing shortages, and lack of teacher training further restrict the ability to deliver comprehensive education at scale [4]. These challenges are compounded by the complexity of implementing multi-level interventions within diverse school environments, where success depends on alignment with existing systems, administrative support, and resource availability [14]. Collectively, this body of evidence demonstrates that logistical infrastructure—not just curriculum content—is a critical barrier to expanding large-scale cancer education efforts in schools.

Philadelphia offers a good case-study example of this. Community-based contexts in the Philadelphia region demonstrate that many schools are in danger of closing due to lack of resources; where schools are struggling to meet basic requirements to deliver literacy and mathematics instruction, recruit teachers, or stay open at all, providing cancer prevention education falls very low on the priority list [6,13]. Even in better resourced schools in the Philadelphia region, such as the school where the intervention in this study took place, constraints on finances, state and Federal curriculum requirements, and teacher resources all contribute to making the implementation of a large-scale health literacy program extremely difficult [6,13]. Needs assessments and direct engagement with families in Philadelphia have documented limited availability of structured, age-appropriate cancer education in under-resourced schools [3,15,16].

The purpose of this pilot study was therefore to evaluate the impact of a smaller-scale school-based cancer education intervention on cancer-related knowledge, health literacy, and risk-reduction awareness among students in grades 5 through 8 at a private school in Pennsylvania. The aim was to discover if a school impacted by typical constraints relating to finances, state and Federal curriculum requirements, and teacher resources would find it feasible and effective to deliver an educational session limited to a single class period and a small group of students.

## 2. Materials and Methods

This study was designed as a pilot study to evaluate the acceptability, implementation, and preliminary effectiveness of a small-scale school-based cancer education intervention. Pilot studies are critical for informing the design of larger, controlled trials by identifying logistical considerations, refining educational content, and assessing initial signals of impact. Given the exploratory nature of this study, a small sample size and single-group pre–post design were considered appropriate.

### 2.1. Sample and Sampling

The study was conducted at a private middle school in Pennsylvania. Participants included 24 students enrolled in grades 5 through 8, divided into two separate groups who each experienced the same intervention separately. Demographic data on the group of students, as seen in Table 1, shows that the sample was diverse in terms of gender, race, ethnicity, religion, and language—all contextual factors that might impact cancer awareness and education in students.

Participation was voluntary, and all students present on the day of instruction were invited to participate. The selected age group reflects a critical developmental stage during which foundational health beliefs, behaviors, and self-advocacy skills are formed [7,17,18].

### 2.2. Intervention

The intervention consisted of two separate 45 min, developmentally appropriate cancer education sessions, delivered by a health professional with clinical oncology experience. For each session, students were in a normal classroom setting, arranged in groups of 4 at their usual classroom desks. Their usual classroom teacher was also present during the session. The educator delivered the intervention via PowerPoint presentation, supplemented heavily with dialogue and discussion with the students. The intervention’s developmental appropriateness was determined through pre-delivery screening and evaluation by the school administration and educators; the school district also handled the acquisition of consent from students’ parents. This format represented a low investment for the school in terms of time, classroom and building resources, and money; it also provided students with a positive and safe learning environment, using materials and settings they were already comfortable with.

Content included cancer biology, modifiable risk factors, genetics, HPV vaccination, and self-advocacy. The lesson materials were designed using information curated from three well-known and reputable cancer-research sources: the CDC, the American Cancer Society, and the University of Texas MD Anderson Cancer Center. Key topics covered within the education session included:

☐ Definition of cancer and abnormal cell growth
☐ Modifiable risk factors (nutrition, physical activity, sun exposure, alcohol, tobacco)
☐ Role of family history, genetics, and genetic testing
☐ Vaccination as cancer risk reduction (HPV)
☐ Body awareness, symptom recognition, and self-advocacy

### 2.3. Research Instrument

A structured assessment tool developed by the researchers based on existing health literacy and cancer education instruments and refined for age-appropriate comprehension was used to measure cancer-related knowledge, risk awareness, and risk reduction understanding. The survey included a combination of multiple-choice, yes/no, and open-ended questions assessing the following domains:

☐ Understanding of what cancer is
☐ Awareness of modifiable cancer risk factors
☐ Knowledge of cancer risk reduction strategies
☐ Awareness of genetic testing and family history
☐ Understanding of vaccination as cancer risk reduction
☐ Recognition of bodily changes and self-advocacy behaviors.

Pre-intervention surveys were administered immediately prior to the session to determine students’ baseline knowledge, and post-intervention surveys were administered immediately afterward to assess initial signals of impact of the education session. To determine feasibility, the researcher acted as instructor during the intervention and took detailed notes about the experience of delivering the intervention in real time, to document challenges, strengths, and areas for improvement.

Quantitative data were analyzed descriptively using frequencies and percentages. Qualitative responses from open-ended questions were reviewed and analyzed thematically to identify patterns in students’ understanding, perceptions, and reflections related to cancer prevention and health awareness. These responses were used to contextualize the quantitative findings and provide insight into the context of students’ learning, their ability to apply the learnings to their lived experiences, areas where the intervention could be strengthened. Given the exploratory nature of the study and small sample size, inferential statistical analyses were not conducted.

## 3. Results

### 3.1. Baseline Characteristics and Knowledge

Pre-intervention assessments revealed substantial gaps in students’ baseline cancer-related knowledge (see Table 2). Only 16% of students were able to accurately define cancer, indicating limited foundational understanding prior to the educational session. Findings from the open-ended questions showed that students demonstrated varying levels of baseline knowledge regarding cancer, cancer risk reduction, clinical trials, informed consent, advocacy, and cancer screening. Awareness of genetic testing, informed consent, HPV vaccination, and clinical trials was limited. While most students reported familiarity with sunscreen, understanding of its role in cancer risk reduction varied, suggesting exposure to fragmented health messaging rather than comprehensive education.

Several open-ended responses reflected limited understanding of cancer and cancer risk reduction concepts prior to the intervention. When asked, “What is cancer?” Students described it as “a sickness,” “an illness,” “a lump in the chest,” and “a disease that can be fatal if not treated.” One particularly noteworthy response stated, “Cancer is a disease people die from.” This statement highlights the fear and fatalistic perceptions that some youth may hold regarding cancer and underscores the importance of providing accurate, age-appropriate cancer education.

Family cancer history was captured to contextualize the connections students’ made between their family history of cancer and their own risk reducing behaviors. Experiences with cancer diagnoses within the family was commonly reported (see Table 3), with 57% of students indicating at least one family member with cancer and an additional 13% reporting multiple relatives with cancer; 26% were unsure. Uncertainty regarding family cancer history among over one-quarter of students highlights gaps in intergenerational health communication and reinforces the need for early education that encourages family dialogue and awareness.

Students also demonstrated varying levels of awareness regarding family cancer history in the open-ended qualitative responses. Responses included, “My grandpa, 80, pancreatic,” and “Grandma, breast, 55–70 I think,” suggesting that some students were aware of cancer diagnoses among relatives but had limited knowledge of hereditary cancer risk and family health history.

Baseline understanding of genetics-related concepts reflected this gap. Awareness of genetic testing was mixed, with 52% reporting having heard of it, while nearly half had no familiarity. Knowledge of cancer screenings demonstrated in open-ended qualitative responses was also more limited at baseline. Several students responded, “I don’t know,” while others incorrectly identified treatments rather than screening methods, such as “Chemo.” Some students identified screening tools including “Mammogram and X-ray,” indicating partial awareness of cancer detection practices. This limited awareness suggests that many students may lack understanding of how inherited risk differs from modifiable risk factors.

Baseline knowledge of clinical trials also varied considerably in the open-ended qualitative responses. While one student stated, “Yes. I do clinical trials for nut allergies,” another responded, “I don’t know,” indicating differing levels of familiarity with research participation and clinical studies. Similarly, students generally associated informed consent with permission and agreement. Student responses included “Agreeing to something,” “Permission to do something,” “Explicitly given permission,” “Approval/saying yes,” and “Making sure the other person is okay with whatever is happening.” These responses suggest a foundational understanding of consent concepts. When asked about advocacy, students commonly responded with phrases such as “Protest,” “Speak up for yourself,” and “Stand up for yourself,” indicating an emerging understanding of self-advocacy and community engagement.

Students were able to demonstrate basic awareness of sun safety and cancer risk reduction in the open-ended qualitative responses. Responses to the question, “What does sunscreen/protection mean to you?” included “SPF!!!,” “Protecting your skin,” “Sunscreen, sun, sweater, sun hat, etc.,” “Stuff that protects you from the sun,” and “Wearing sunscreen so you don’t get cancer.” These responses suggest that many students were able to connect sunscreen use with cancer risk reduction post intervention.

Dietary intake and physical activity questions were included to characterize baseline health behaviors rather than to assess post-intervention change (see Table 2). Given the brief, single-session nature of the intervention, changes in meal frequency, dietary patterns (including plant-forward eating), or physical activity were not expected between the pre- and post-tests. These measures were used to contextualize students lived experiences, family environments, and health behaviors and to inform interpretation of knowledge-related outcomes related to cancer risk awareness and prevention.

Baseline dietary patterns reflected inconsistent nutrition behaviors. Forty-two percent of students reported that they typically do not eat breakfast. Among those who did eat breakfast, commonly reported foods included cereal (42%), eggs (38%), fruit (33%), meat (29%), toast or bread (21%), and pastries (21%). Lunch patterns were similarly inconsistent; although students reported consuming foods such as pasta or rice (54%), sandwiches (46%), meat (42%), vegetables (33%), salads (25%), fruit (21%), and dairy products (17%), 85% indicated that they do not usually eat lunch. In contrast, all students reported eating dinner, most commonly pasta or rice (87%), meat (78%), vegetables (65%), and salads (43%). These findings suggest irregular meal patterns that may influence long-term health behaviors and risk trajectories.

Physical activity habits varied across participants. More than half of students (54%) participated in team sports such as soccer, basketball, or volleyball. Other reported activities included cardiovascular exercise (29%), participation in multiple forms of activity (38%), yoga or Pilates (17%), outdoor activities (17%), strength training (13%), and group fitness classes (8%). Eight percent reported not engaging in regular physical activity.

Data were also collected on students’ support networks and levels of agency to contextualize the systemic factors this might impact students’ willingness and likelihood to advocate for their own health (see Table 4). Most students (71%) reported having health insurance, while 25% were unsure and 4% preferred not to respond. All students (100%) reported feeling supported by family or friends, indicating strong perceived social support despite gaps in formal health knowledge. When asked how they recognize when something is wrong with their health, students cited paying attention to physical symptoms (51%), trusting their instincts (21%), seeking medical advice (21%), educating themselves (13%), and attending routine checkups (13%); however, 21% reported uncertainty. Several students indicated that their cancer-related knowledge came from informal or fragmented sources, describing learning “bits and pieces” rather than receiving structured education, consistent with prior research [2].

### 3.2. Post-Intervention Outcomes

Post-intervention assessments demonstrated marked improvements across multiple domains of cancer-related knowledge and awareness (see Table 5). All students (100%) were able to correctly define cancer following the educational session, representing a substantial increase from baseline. Awareness of smoking- and alcohol-related cancer risks increased to 96%, and understanding of drug- and substance-related cancer risks increased to 91%. Knowledge of the preventive role of sunscreen also improved, increasing to 90%.

Post-intervention qualitative open-ended responses also suggested improved understanding of baseline knowledge regarding cancer, cancer risk reduction, clinical trials, informed consent, advocacy, and cancer screening, supporting the quantitative findings of increased knowledge following participation in the educational session.

Notably, understanding of genetics and familial cancer risk improved following the intervention. Awareness of genetic testing increased to 83% post-intervention, compared with 52% at baseline. This gain is particularly meaningful given that 70% of students reported a known family history of cancer or uncertainty regarding familial cancer diagnoses.

Knowledge of the HPV vaccine also improved substantially, with 57% of students correctly identifying its purpose, compared with 21% on the pre-test. An additional 35% reported having heard of the vaccine but remained unsure of its purpose, while 22% reported no awareness. These findings suggest that early education may initiate awareness that can be reinforced through future healthcare encounters and caregiver engagement.

Understanding of clinical trials increased from 30% at baseline to 60% following the intervention, reflecting improved comprehension of research participation and medical advancement. Understanding of informed consent also improved slightly, with 86% demonstrating clear comprehension post-test, reinforcing students’ growing awareness of autonomy and patient rights within healthcare settings.

Students demonstrated improved health awareness and self-advocacy following the intervention. Post-test responses indicated that 74% would pay attention to physical symptoms, 30% would trust their instincts, 26% would seek medical advice, 26% would educate themselves on health concerns, and 22% would monitor their health through screenings or routine checkups. Only 4% reported uncertainty about recognizing when something is wrong with their health, suggesting increased confidence in identifying potential warning signs.

In their qualitative open-ended responses, when asked about cancer screenings, students correctly identified examples such as “Mammogram.” Responses regarding sunscreen and risk reduction also reflected stronger understanding, with one student stating, “I think it means to always use sunscreen!” When asked, “What is cancer?” Post-intervention responses were more concise and accurate, including “A disease.”

In contrast, understanding of advocacy decreased from 96% at baseline to 60% post-intervention. This decline may reflect differences in question interpretation or increased awareness of the complexity of advocacy following instruction. Despite this decrease, understanding of cancer itself improved substantially, with all students providing a correct definition post-test. When asked about breast cancer screening methods, 30% of students were able to identify them, while 70% reported uncertainty or inability to recall the information, indicating the need for reinforcement and repetition in future educational efforts.

## 4. Discussion

### 4.1. Initial Signals of Impact

This pilot study demonstrates that a small-scale brief, school-based cancer education intervention can significantly improve cancer-related knowledge, genetic literacy, and risk reduction awareness among middle school students. Post-intervention improvements across all knowledge domains suggest that even a single educational session can meaningfully improve cancer literacy.

The magnitude of knowledge gains observed across multiple domains—including cancer definition, modifiable risk factors, genetic testing, vaccination, and clinical research—highlights the effectiveness of even small-scale developmentally appropriate education delivered in a structured school setting. These findings are consistent with national evidence showing that early health education can improve disease-related knowledge and shape long-term health beliefs during adolescence [1,7,17,19]. This study demonstrates that it is feasible and effective for such early education interventions to take the form of a small-scale, two separate session, interventions with a small group of students.

Baseline assessments revealed substantial gaps in foundational cancer knowledge, despite frequent exposure to cancer through family history. More than half of participants reported at least one family member with cancer, yet many students were unable to accurately define cancer or distinguish between inherited and modifiable risk factors. This finding aligns with prior research indicating that experiential exposure alone does not reliably translate into accurate health literacy and may, in some cases, reinforce misconceptions or fatalistic beliefs [1,3,4,20,21]. This study therefore confirms that there is a clear need for interventions that provide students with clear, research-based oncology knowledge.

The limited baseline understanding of genetic testing, HPV vaccination, and clinical trials further reflects broader national patterns. Studies of adolescent and adult populations consistently demonstrate low awareness of cancer risk reduction strategies and confusion surrounding genetic risk [3,4,5]. Without explicit instruction, students may overestimate the role of genetics in cancer causation, underestimate the importance of lifestyle factors, and perceive cancer as largely unavoidable [1,4]. Addressing these misconceptions early is critical for supporting balanced risk perception and empowering youth to engage in risk reducing behaviors and health advocacy. The baseline findings in this study underscore the importance of providing students with empowering risk reducing knowledge as early as possible through flexible, easy-to-implement interventions.

Importantly, this study was not designed to produce immediate behavior change. Instead, it targeted cognitive precursors—knowledge, awareness, and perceived relevance—that are necessary for later behavioral adoption [7,17]. National models of behavior change emphasize that understanding risk, recognizing personal relevance, and developing self-efficacy precede sustained behavioral modification [7,17]. By increasing students’ understanding of how nutrition, physical activity, sun exposure, alcohol, and tobacco influence cancer risk, this intervention laid foundational groundwork for future engagement in health-promoting behaviors. The observed gains in awareness of health-seeking behaviors and symptom recognition after only a single educational session further suggest improved readiness to engage with healthcare systems as students age.

Improved genetic literacy represents a particularly meaningful outcome of this intervention. Post-intervention data indicate increased understanding of genetic testing and family history as components of cancer risk. Clarifying that inherited factors account for a minority of cancer cases—while still holding significance for individual susceptibility—may help reduce fatalism and promote proactive risk reduction behaviors [1,4]. Enhanced genetic literacy may also facilitate family-level health communication. Adolescents who understand the relevance of family health history may be better positioned to ask questions, engage in informed discussions with caregivers, and recognize the importance of screening and preventive care.

First, while overall knowledge increased significantly, inconsistent retention across topics (e.g., decreased understanding of advocacy, certainty about vaccination) indicates a need for deeper emphasis on key concepts rather than broad coverage. The observed decline in understanding of advocacy post-intervention in this study in particular warrants careful consideration. This shift may reflect increased awareness of the complexity of health advocacy or confusion between general civic advocacy and health-specific self-advocacy terminology. As students gained more detailed information about cancer and healthcare systems, their confidence in defining advocacy may have become more nuanced. Future interventions should prioritize fewer topics with greater depth, particularly those that students struggled to retain, such as advocacy and self-advocacy (clarifying meaning and real-life application), and HPV vaccination (including why it matters for cancer risk reduction). Future interventions may benefit from clearer framing, applied examples, and repeated reinforcement of health-specific advocacy concepts to support sustained comprehension.

Second, students’ understanding of concepts such as informed consent, genetic testing, and clinical trials improved, but not to the extent the researcher hoped. The lower-than-expected results in some domains (ex. HPV vaccination, informed consent, and self-advocacy) indicate that passive learning alone may not be sufficient, particularly for more complex topics. These findings highlight the importance of reinforcing complex or abstract concepts through repetition and application. Future interventions may benefit from real-life scenarios or case-based examples, interactive discussions that connect concepts to students’ lived experiences, hands-on demonstrations or visual tools, and age-appropriate storytelling to improve relatability and understanding.

Third, baseline data revealed gaps in lifestyle knowledge and behaviors (e.g., skipped meals, limited understanding of diet-cancer links) that were more extensive than the researchers expected. This suggests the need to more explicitly integrate nutrition, physical activity, and environmental risk factors into the curriculum. While these were included in the pilot study, they could be strengthened in future interventions by making clearer connections between daily habits and long-term cancer risk; including practical, actionable strategies (e.g., “what a balanced day of eating looks like”); and addressing social determinants of health in an age-appropriate way.

Demographic responses in this study indicated varied household composition and a high prevalence of family exposure to cancer, which is significant because household composition and family experiences may further impact cancer learning. Prior research suggests that family conversations about health are associated with improved awareness and preventive behaviors across generations [4,17]. Meaningful cancer risk reduction cannot occur in isolation from the environments in which children live. Furthermore, young people often lack agency to implement preventative behaviors in their home life, with decisions about health care, food, activities, and so forth usually being controlled by adults [7,22]. Students with siblings may benefit from informal peer-to-peer learning within the home, as information introduced in the classroom is discussed, modeled, or reinforced through sibling interactions. Furthermore, caregivers play a central role in shaping dietary behaviors, physical activity opportunities, healthcare access, and health-related decision-making. Without caregiver understanding, student knowledge may remain abstract or unsupported by daily practices. With this in mind, future interventions should include both students and their caretakers in the conversation about health and cancer, through shared assignments, take-home materials, and encouragements for student-led conversations at home, all of which can further help reduce risks.

### 4.2. Considerations for Future Implementation

The findings also point to several ways the feasibility of the intervention can be improved to maximize effectiveness and scalability. First, the study facilitator noted variability due to group dynamics across the two participant groups, suggesting the need for more standardized delivery methods. This could include a structured facilitator guide to ensure consistency; standardized materials, such as slides, activities, or discussion prompts; and training for educators or facilitators to maintain fidelity across settings.

Second, given the diversity of the student population, there is an opportunity to enhance delivery through culturally responsive and inclusive approaches, such as incorporating diverse representations in examples and materials; acknowledging varying family, cultural, and health experiences; and providing multilingual or adaptable resources where appropriate.

Finally, mental health considerations are essential when introducing sensitive topics such as cancer in school settings [6,9,11,23]. While some universal school-based mental health interventions have been associated with increased distress in certain subgroups [9,11], this intervention was educational rather than therapeutic in nature. Content in this study emphasized empowerment, body awareness, and informed decision-making without requiring emotional disclosure. When sensitive topics are presented in an age-appropriate, non-alarmist manner, education may reduce fear by increasing understanding and perceived control [7,17]. Although this was not intentionally designed into the intervention, the facilitator noticed that it became a necessity on the day to help students feel safe. Qualitative responses in this pilot study suggest students benefit from emotionally and socially relevant framing. By incorporating opportunities for family and community connections, personal storytelling or survivor narratives, and reflection, future facilitators may be able to create a safe environment to support complex and emotional oncology conversations with students.

### 4.3. Implications for Public Health and Equity

Findings from this study suggest that cancer education can be feasibly and effectively integrated into school health curricula as a risk reduction-focused public health strategy at a small scale and utilizing limited resources. Even a brief developmentally appropriate educational intervention produced meaningful gains in cancer knowledge, risk awareness, and self-advocacy among middle school students. These findings support the inclusion of small-scale cancer prevention education initiatives as a core component of comprehensive school health programming, rather than an optional or extracurricular topic.

From a practice perspective, school nurses, health educators, counselors, and allied school health professionals are uniquely positioned to deliver cancer education in alignment with existing instruction on nutrition, physical activity, sun safety, vaccination, and general wellness. Integrating cancer education within these established domains may reduce instructional burden while reinforcing consistent health messaging.

The findings from this study also have implications for education beyond the classroom. Findings in this study indicated that much of students’ cancer-related knowledge came from informal or fragmented sources. It is important to remember that adolescents often lack autonomy over preventative measures such as dietary choices, physical activity opportunities, and healthcare access, making knowledge acquisition a particularly critical first step toward risk reduction [7]. Teaching caregivers about cancer risk factors, genetics, screening, informed consent, and prevention strategies may help ensure that messages delivered in school are reinforced at home and accurately interpreted. This dynamic may extend the reach of school-based education beyond the individual student, amplifying its potential public health impact [17]. Caregiver education also strengthens the broader patient–provider relationship across the life course. When families understand cancer-related concepts and prevention language, they are better positioned to engage in informed conversations with healthcare providers, ask relevant questions, consent to recommended screenings or vaccinations, and advocate effectively for themselves and their children. This shared health literacy promotes trust, communication, and continuity of care—key components of effective cancer risk reduction and early detection. The small-scale and flexible nature of the intervention in this study offers intriguing possibilities for learning outside of the classroom, by offering knowledge in a bite-sized format that is easy to transfer from the classroom to the home. As already discussed, interventions could be made even stronger by directly including guidance for extending cancer learning outside the classroom.

Overall, the findings in this pilot study underscore the feasibility and effectiveness of shifting school health education frameworks toward small-scale prevention-oriented models that emphasize long-term risk reduction. If these findings can be replicated in larger-scale studies, it is reasonable to argue that policymakers and educational leaders should prioritize funding and structural support for training school-based health professionals in delivering small-scale, low-resource cancer prevention education in a flexible and easy-to-implement format. Investment in professional development that integrates trauma-informed, culturally responsive, and developmentally appropriate approaches with existing health education may allow schools to deliver cancer education to shape student health literacy in the classroom and beyond. To support these long-term goals, future research should include larger, more diverse samples across multiple school settings and incorporate control or comparison groups. Longitudinal studies are needed to evaluate knowledge retention and behavioral outcomes over time.

### 4.4. Limitations

This study should be interpreted within the context of its pilot design. The small sample size (*n* = 24) and two separate groups-site setting limit generalizability but are consistent with feasibility-focused research. The short duration of the intervention and immediate post-test assessment preclude evaluation of long-term knowledge retention or behavioral change. The study assessed short-term changes in knowledge rather than long-term knowledge retention or behavior modification. Dietary intake and physical activity measures were descriptive and intended to establish baseline context; changes in these behaviors were not evaluated as outcomes. In this study, determining effectiveness on such a small scale was a crucial component of the study objective.

The absence of a control group limits causal inference; however, the pre–post design provides preliminary evidence of intervention impact. Logistical constraints required the intervention to be delivered across two instructional sessions, which may have introduced variability in engagement and knowledge retention due to differences in group dynamics. Additionally, some participants provided incomplete or partial responses, which may have reduced the precision of certain findings. Student interest and engagement also varied. Some students demonstrated limited interest in the topic, which may have influenced attention, participation, and comprehension during the lesson. Variability in developmental readiness and personal relevance of cancer education among middle school students may have further affected engagement levels and learning outcomes. Future longitudinal studies with larger, more diverse samples across multiple school settings and incorporating control or comparison groups could mitigate these limitations.

Geographic and environmental exposures were not assessed, limiting insight into contextual cancer risk factors such as pollution, neighborhood resources, or access to healthcare. Furthermore, the study focused on general cancer awareness and risk reduction strategies related primarily to adult-onset cancers. Pediatric cancers, which are often biologically distinct and frequently driven by genetic, developmental, or prenatal factors, were not specifically addressed. Furthermore, this study was conducted within a private school with small class sizes. While this location offered the benefit of enabling students and instructions to engage in rich discussions with fewer distractions, additional studies are needed to determine whether the same results could be replicated in public schools with larger class sizes.

A further limitation of the study is that the assessment surveys were developed by the researchers themselves. Although this survey drew on both extensive secondary literature on cancer education and the researchers’ own professional expertise in cancer education, relying on a self-developed instrument rather than a published and validated one may have introduced biases. In future studies, the researchers recommend a collaborative approach to instrument development that draws on the expertise of students and other educators as well as the researchers to minimize introduced bias.

Finally, while cancer risk reduction education emphasizes modifiable risk factors, cancer development is multifactorial and not fully preventable. Many cancers, particularly pediatric malignancies—are not attributable to lifestyle behaviors alone. These limitations highlight the importance of framing cancer education within a broader context that includes genetic, environmental, epigenetic, and social determinants of health.

## 5. Conclusions

This study demonstrates that a brief, developmentally appropriate, school-based cancer education intervention can substantially improve cancer-related knowledge, genetic literacy, and prevention awareness among middle school students. Despite frequent exposure to cancer through family history, baseline assessments revealed significant gaps in understanding of cancer etiology, hereditary risk, prevention strategies, and health system concepts—highlighting that experiential exposure alone does not ensure accurate or actionable knowledge [1,3,4].

The findings reinforce national evidence that because most cancers are driven by modifiable factors rather than inherited risk alone, early education may play a critical role in shaping balanced risk perception [1,2,4]. By explicitly distinguishing genetic susceptibility from behavioral and environmental contributors, school-based education may reduce fatalistic beliefs and support engagement in risk reduction behaviors and health advocacy across the life course [1,7,17,24,25]. By delivering this type of school-based education on a small scale using limited resources, this study has demonstrated that gains in awareness and risk reducing knowledge and behavior can be achieved even where large-scale cancer education is not feasible within the curriculum.

Importantly, this intervention was designed to influence cognitive precursors to behavior change rather than immediate behavioral outcomes. Knowledge acquisition represents a necessary foundation for later risk reduction, particularly for youth who may lack autonomy or resources to modify behaviors independently [7,17,26]. Improved genetic literacy and prevention awareness may also facilitate family-level conversations about health history, screening, and informed decision-making.

From a public health and equity perspective, the identification of substantial baseline knowledge gaps within a private school setting suggests that deficiencies in foundational cancer education are likely widespread. Students in under-resourced schools—who often face limited access to preventive health information and healthcare services—may experience even greater disparities [1,4,27]. Embedding cancer education into standard school health curricula in the form of small-scale, low-resource, flexible educational sessions represents a feasible, scalable, and potentially cost-effective strategy for improving health literacy and reducing future cancer burden.

Collectively, these findings support small-scale, early, developmentally appropriate cancer education as a critical component of comprehensive cancer risk reduction efforts. Integrating prevention-focused cancer education into schools using this format may help overcome resource barriers, allowing for cancer education that can normalize conversations about cancer, empower students with accurate information, and lay the groundwork for sustained risk reduction across the lifespan.

## Figures and Tables

**Table 1:** Participant demographic characteristics.

Characteristic	Category	Percentage (%)
Country of origin	data	100%
Race	White/Caucasian	50%
	Black/African American	21%
	Asian	8%
	Native American	0%
	Pacific Islander	0%
	Mixed/Multicultural	13%
	Other	13%
Ethnicity	African	17%
	Asian	8%
	Middle Eastern	8%
	Hispanic/Latinx	4%
	Other	13%
	Prefer not to say	4%
	White/European	50%
Gender	Male	29%
	Female	67%
	Non-binary	4%
Primary language	English	100%
Secondary language	Spanish	55%
	Arabic	5%
	Japanese	10%
	French	5%
	Other (Patois)	5%
Religion	Christianity	35%
	Judaism	17%
	Islam	4%
	Hinduism	4%
	Buddhism	4%
	Atheism/Agnosticism	9%
	Other	17%
	Prefer not to say	17%

Note: Some participants selected more than one response for the categories of race, ethnicity, and religion. Some participants did not select a response for the category of secondary language.

**Table 2:** Baseline risk-reducing health behaviors.

Preventative Behavior	Students Reporting (%)
Eat breakfast	58%
Eat lunch	15%
Eat dinner	100%
Participate in team sports	54%
Other physical activity	46%

**Table 3:** Family cancer history.

Category	Students (%)
At least one family member with cancer	57%
Multiple relatives with cancer	13%
Unsure	30%

**Table 4:** Students’ support networks and levels of agency.

Category	Students (%)
Has health insurance	71%
Feel Supported by family and friends	100%
Pay attention to physical symptoms	51%
Trust their own instincts	21%
Willing to seek medical advice	21%
Willing to educate themself	13%
Attend routine checkups	13%

**Table 5:** Intervention pre- and post-test results indicating changes in student knowledge and awareness of cancer.

Domain	Pre-Test Correct (%)	Post-Test Correct (%)
Define cancer	16%	100%
Smoking/alcohol risk	45%	96%
Sunscreen/UV protection	60%	90%
Genetic testing/family risk	52%	83%
HPV vaccination	21%	57%
Clinical trials	30%	60%
Informed consent	70%	86%
Symptom recognition/self-advocacy	36%	52%

## Data Availability

Data is available from the corresponding author upon reasonable request.

## References

[1] Abraham O., Szela L., Feng E., Egbujor M., Gay S. (2023). Exploring youth perceptions about cancer prevention and preferences for education: A qualitative study. J. Cancer Educ..

[2] American Cancer Society (2024). Cancer Facts & Figures 2024.

[3] Guzman-Holst C., Streckfuss Davis R., Andrews J.L., Foulkes L. (2025). Potential harm from school-based group mental health interventions: A scoping review. Child Adolesc. Ment. Health.

[4] McCutchan G.M., Wood F., Edwards A., Richards R., Brain K.E. (2015). Influences of cancer symptom knowledge, beliefs and barriers on cancer symptom presentation in relation to socioeconomic deprivation: A systematic review. BMC Cancer.

[5] Foulkes L., Andrews J.L., Reardon T., Stringaris A. (2023). Measuring and reporting potential harm from universal school-based mental health interventions: Research recommendations for an ethical issue. PsyArXiv.

[6] Anbazhagan S., Shanbhag D., Antony A., Bhanuprakash K., Anbazhagan S., Chandran N., Ramakrishna G. (2016). Comparison of effectiveness of two methods of health education on cancer awareness among adolescent school children in a rural area of Southern India. J. Fam. Med. Prim. Care.

[7] Miller E.J., Crane C., Medlicott E., Robson J., Taylor L. (2023). Non-positive experiences encountered by pupils during participation in a mindfulness-informed school-based intervention. Sch. Ment. Health.

[8] Wigelsworth M., Squires G., Birchinall E., Kalambouka A., Lendrum L., Black L., Troncoso P., Troncoso J., Troncoso E., Britteon P. (2018). FRIENDS for Life: Evaluation Report and Executive Summary.

[9] Montero-Marin J., Allwood M., Ball S., Crane C., De Wilde K., Hinze V., Jones B., Lord L., Nuthall E., Raja A. (2022). School-based mindfulness training in early adolescence: What works, for whom, and how in the MYRIAD trial. Evid. Based Ment. Health.

[10] Stallard P., Sayal K., Phillips R., Taylor J.A., Spears M., Anderson R., Araya R., Lewis G., Millings A., Montgomery A.A. (2012). Classroom-based cognitive behavioural therapy in reducing symptoms of depression in high-risk adolescents: A pragmatic cluster randomised controlled trial. BMJ.

[11] Jassem J., Ozmen V., Bacanu F., Drobniene M., Eglitis J., Lakshmaiah K.C., Kahan Z., Mardiak J., Pieńkowski T., Semiglazova T. (2013). Delays in diagnosis and treatment of breast cancer: A multinational analysis. Eur. J. Public Health.

[12] Kirnan J., Fotinos G., Pitt K., Lloyd G. (2025). School-based mental health education: Program effectiveness and trends in help-seeking. Int. J. Environ. Res. Public Health.

[13] Vinson C., Villalobos A., Correa-Mendez M., Neta G. (2025). An analysis of National Cancer Institute-funded scale-up research. Front. Health Serv..

[14] Schuler B.R., Saksvig B.I., Nduka J., Beckerman S., Jaspers L., Black M.M., Hager E.R. (2018). Barriers and enablers to the implementation of school wellness policies: An economic perspective. Health Promot. Pract..

[15] Lawrence S., FitzGerald S., Hegarty J., Saab M.M. (2025). Cancer awareness among adolescents in second-level education: A mixed-methods systematic review. Health Educ. Res..

[16] Seely H.D., Gaskins J., Pössel P., Hautzinger M. (2023). Comprehensive prevention: Evaluation of peripheral outcomes of a school-based prevention program. Res. Child Adolesc. Psychopathol..

[17] Bailey S., Grummitt L., Birrell L., Kelly E., Gardner L., Champion K., Chapman C., Teesson M., Barrett E., Newton N. (2023). Young people’s evaluation of an online mental health prevention program for secondary school students: A mixed-methods formative study. Ment. Health Prev..

[18] American Institute for Cancer Research (2019). Cancer Risk Awareness Survey.

[19] Darlington E.J., Violon N., Jourdan D. (2018). Implementation of health promotion programmes in schools: An approach to understand the influence of contextual factors on the process. BMC Public Health.

[20] da Silva D.B., Pianovski M.A.D., da Costa M.T.F. (2024). Childhood and adolescent cancer: Early diagnosis challenges. Rev. Assoc. Med. Bras..

[21] Fares Sabik E. (2026). Genetic basis of familial cancer risk: A narrative review. DNA.

[22] Fraser G.E., Butler F.M., Shavlik D.J., Mathew R.O., Oh J., Sirirat R., Abramov D., Sveen L.E. (2025). Longitudinal associations between vegetarian dietary habits and site-specific cancers in the Adventist Health Study-2 North American cohort. Am. J. Clin. Nutr..

[23] Mackenzie K., Williams C. (2018). Universal, school-based interventions to promote mental and emotional well-being: What is being done in the UK and does it work? A systematic review. BMJ Open.

[24] National Cancer Institute, Division of Cancer Control and Population Sciences (2024). Investments in Cancer Control Research: 2024 Overview and Highlights.

[25] Sanchez J.I., Adjei B.A., Randhawa G., Medel J., Doose M., Oh A., Jacobsen P.B. (2022). National Cancer Institute–funded social risk research in cancer care delivery: Opportunities for future research. JNCI J. Natl. Cancer Inst..

[26] Chen M., Ramchandani N. (2026). Missing the malignancy in primary care: A case study on pediatric osteosarcoma. J. Pediatr. Health Care.

[27] World Health Organization (2023). Cancer Prevention and Control.

